# Overview of Bile Acids Signaling and Perspective on the Signal of Ursodeoxycholic Acid, the Most Hydrophilic Bile Acid, in the Heart

**DOI:** 10.3390/biom8040159

**Published:** 2018-11-27

**Authors:** Noorul Izzati Hanafi, Anis Syamimi Mohamed, Siti Hamimah Sheikh Abdul Kadir, Mohd Hafiz Dzarfan Othman

**Affiliations:** 1 Institute of Medical Molecular Biotechnology, Faculty of Medicine, Universiti Teknologi MARA, Sungai Buloh 47000, Selangor, Malaysia; nih220688@yahoo.com (N.I.H.); mizsyamimi@gmail.com (A.S.M.); 2 Department of Biochemistry and Molecular Medicine, Faculty of Medicine, Universiti Teknologi MARA, Sungai Buloh 47000, Selangor, Malaysia; 3 Advanced Membrane Technology Research Centre (AMTEC), Universiti Teknologi Malaysia, Johor Bharu 81310, Johor, Malaysia; hafiz@petroleum.utm.my

**Keywords:** heart, bile acid, ursodeoxycholic acid, cardioprotection, signaling

## Abstract

Bile acids (BA) are classically known as an important agent in lipid absorption and cholesterol metabolism. Nowadays, their role in glucose regulation and energy homeostasis are widely reported. BAs are involved in various cellular signaling pathways, such as protein kinase cascades, cyclic AMP (cAMP) synthesis, and calcium mobilization. They are ligands for several nuclear hormone receptors, including farnesoid X-receptor (FXR). Recently, BAs have been shown to bind to muscarinic receptor and Takeda G-protein-coupled receptor 5 (TGR5), both G-protein-coupled receptor (GPCR), independent of the nuclear hormone receptors. Moreover, BA signals have also been elucidated in other nonclassical BA pathways, such as sphingosine-1-posphate and BK (large conductance calcium- and voltage activated potassium) channels. Hydrophobic BAs have been proven to affect heart rate and its contraction. Elevated BAs are associated with arrhythmias in adults and fetal heart, and altered ratios of primary and secondary bile acid are reported in chronic heart failure patients. Meanwhile, in patients with liver cirrhosis, cardiac dysfunction has been strongly linked to the increase in serum bile acid concentrations. In contrast, the most hydrophilic BA, known as ursodeoxycholic acid (UDCA), has been found to be beneficial in improving peripheral blood flow in chronic heart failure patients and in protecting the heart against reperfusion injury. This review provides an overview of BA signaling, with the main emphasis on past and present perspectives on UDCA signals in the heart.

## 1. Introduction

**Figure 1:**
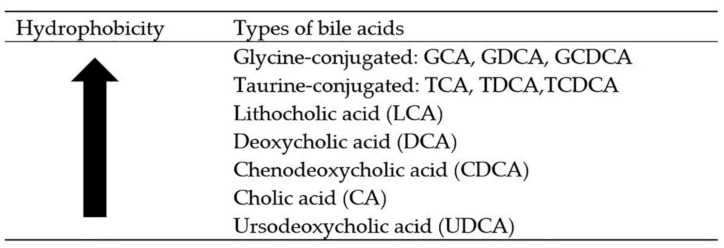
The hydrophobicity of bile acids decrease with increase in OH groups. (GCA, glycocholic acid; GDCA, glycodeoxycholic acid; GCDCA, glycochenodeoxycholic acid; TCA, taurocholic acid; TDCA, taurodeoxycholic acid; TCDCA, taurochenodeoxycholic acid).

Bile acids (BAs) are derived from cholesterol and are stored in the gall bladder. Cholic acid (CA) and chenodeoxycholic acid (CDCA) are the two most common types of primary bile acids, which are synthesized in the liver and conjugated to either taurine or glycine. The conjugated primary bile acids are known as glycocholic acid, taurocholic acid, glycochenodeoxycholic acid (GDCA), and taurochenodeoxycholic acid (TDCA). These conjugated bile acids are known as bile salt. The liver consists of only 2.5–5.0 g of the bile acid pool [1], with 95% of the pool is from bile acid reabsorption occurring in the small intestine [2]. The most common secondary bile acids—deoxycholic acid (DCA) and lithocholic acid (LCA)—are synthesized by microbial flora of the small intestine. These two bile acids are produced as a result of deconjugation and dehydroxylation of primary bile acids. The cytotoxicity of bile acids is dependent on their structural formation, while their hydrophobicity is based on the number and position of the hydroxyl group in the ring structure (Figure 1). Increased serum levels of hydrophobic bile acids, such as CDCA and DCA, have been associated with colon cancer, gallstones, and other gastrointestinal (GI) diseases [3]. Lithocholic acid is the most hydrophobic bile acid, hence, only a small amount of LCA is reabsorbed back into the enterohepatic circulation. Consequently, the amount of LCA in the feces is higher. The least toxic bile acid is called ursodeoxycholic acid (UDCA). Ursodeoxycholic acid is synthesized by dehydroxylation of free bile acid CDCA. It is the most hydrophilic bile acid, and small amounts of it can be found in the feces.

The BA pool circulates in humans through enterohepatic circulation. This enterohepatic circulation involves liver as the main organ, gallbladder as a storage organ for the bile, and the small intestine to help in digestion of fats as well as excretion and reabsorption. Bile acids are synthesized in the liver through enzymatic reactions, where the hydrophobic ring structure of the cholesterol is digested into more water-soluble amphipathic compounds [4]. Four types of transports are involved in bile acid reabsorption: active transport, passive ionic diffusion, passive nonionic diffusion, and passive micellar diffusion. Bile acids are absorbed through these transport systems and circulated back to the liver. Small quantities of bile acids are present in the systemic circulation, and very small amounts are excreted through urine and feces (300–600 mg/day) [5,6].

Classically, bile acids are known to be involved in lipid metabolism [7,8], cholesterol elimination, bile flow, and cholesterol biosynthesis. BAs are now widely reported to play an important role as a signaling molecule in cell proliferation, metabolism, and differentiation of cells such as hepatocytes [9], gastric cancer cells [10], and colon cancer cells [11]. In the past 30 years, more tissues and organs have been shown to be directly or indirectly influenced by BA signals. Hence, our review aims to give an overview of general bile acids signaling with an emphasis on the most hydrophilic BA signals in the heart.

## 2. Bile Acid as a Signaling Molecule

In the last decade, nuclear receptor-mediated response (farnesoid X-receptor, FXR) was discovered as the key receptor of BA signal [12]. About 10 years after FXR discovery, membrane receptor-mediated response (Takeda G-protein-coupled receptor 5, TGR5) was reported as the first G-protein-coupled receptor specific for BAs [13]. In addition, muscarinic receptor and sphingosine-1-phosphate (S1P) are the other types of receptors reported in mediating the signal of BAs. A few studies have suggested an involvement of other nonclassical routes of BA signal, such as large conductance voltage- and Ca^2+^-activated potassium (K^+^) (BK) channels. This section gives an overview of BA signal reported by studies using in vivo and in vitro models as well as clinical studies reported in the past 30 years (Table 1).

### 2.1. Nuclear Receptor-Mediated Response

Farnesoid X-receptor FXR) is the prominent target for most BAs. FXR ligand-binding domain of hydrophobic face binds to hydrophilic face of BAs. The function of FXR receptor was first described in the gut to aid the reabsorption of bile acids from the small intestine to the portal system. FXR is known to be highly expressed in the liver, small intestine, adipose tissue, pancreas, and adrenals [32]. FXR was first reported to be expressed in normal vascular smooth muscle and atherosclerotic blood vessels of humans by Bishop-Bailey et al. [33]. The group further reported that FXR regulates cell proliferation and activates expression of FXR target genes, small heterodimer partner (SHP), and the phospholipid transfer protein (PLTP) in vascular smooth muscle cells [33]. Chenodeoxycholic acid is the most prominent FXR bile acid ligand. In LCA-induced cholestasis model, CDCA activity was shown to reduce and bile salt export pump (BSEP) expression was downregulated. This resulted in a decrease in hepatic bile acid secretion and an increase in bile acid concentration in the liver, which might lead to liver damage [34]. This shows that FXR-activated BSEP expression by CDCA is crucial in protecting the liver. CDCA activates FXR expression in the intestine and concurrently activates the intestinal acid-binding protein (IBABP), which mediates cholesterol secretion from the body [35]. Most studies have suggested that FXR regulates lipid and glucose synthesis in most cells involved in energy homeostasis [36]. In the liver, an increased level of BA activates FXR and upregulates cholesterol 7α hydroxylase (CYP7A1) activity to increase conversion of cholesterol to BA [37]. CYP7A1 is a rate-limiting enzyme for bile acid synthesis. FXR is activated most effectively by CDCA in comparison to DCA, LCA, and CA. In cholangiocytes, unconjugated CDCA has been reported to inhibit tumor proliferation mediated by FXR expression [38]. Recently, Desai et al. [39] reported that excess BAs lead to cardiomyopathy and heart dysfunction. The study reported that in FXR and SHP double knockout mice (model for cirrhosis), reduction in fatty acid metabolism was observed. Consequently, the mice developed cardiac dysfunction due to suppression of *proliferator-activated receptor-γ co-activator 1α* (*PPAR-1α*) gene, a key regulator for fatty acid metabolism. This proves that FXR’s role in regulation of the BA level is important for not just the liver function but also for other vital organs, such as the heart.

Apart from the FXR, other nuclear receptors involved in the regulation of lipid and glucose metabolism are pregnane X-receptor (PXR) and Vitamin D receptor (VDR) [40]. In response to environmental molecules, mammals are equipped with a defensive network mediated by xenobiotic receptors, such as the PXR. Pregnane X-receptor works by inducing the expression genes and proteins for xenobiotic metabolism, particularly in the liver and intestine. PXR are also involved in the breakdown and elimination of BAs. Lithocholic acid activates PXR and subsequently upregulates drug resistance protein expressions, such as multidrug resistance protein 1 (MDR1), multidrug resistance-associated protein 3 (MRP3), multidrug resistance-associated protein 2 (MRP2), and cytochrome P450 family 3 subfamily A (CYP3A) for bile acid transport and detoxification [41]. Both FXR and PXR are known to play an important role in eliminating the effect of BAS-induced toxicity by downregulating the expression of CYP7A1 [42]. In an animal study, PXR activation was shown to regulate lipid and energy metabolism, which consequently prevented obesity induced by high-fat diet as well as insulin resistance [43]. These studies demonstrated the important function of PXR in maintaining lipid metabolism.

Vitamin D receptor is activated by its natural ligands, 1α, 25-dihydroxy-vitamin D3 (1α, 25 (OH)_2_- D3) and LCA. Han et al. [44] reported that both ligands activate VDR-signaling pathway by extracellular signal-regulated kinase (ERK) 1/2 and as consequence, VDR is phosphorylated and translocalized into the nucleus. This leads to the inhibition of CYP7A1 gene transcription in human hepatocytes, thus protecting the cells from further damage in cholestatic liver injury [44]. In the small intestine, LCA induces CYP24a1 expression, the VDR target gene, as efficiently as the main natural ligand of VDR, i.e., 1α, 25 (OH)_2_ [45]. The effect is prominently observed in the ileum than jejunum and duodenum. Vitamin D receptor is known as an important nuclear receptor in regulating calcium homeostasis, immunity, and cellular differentiations [46]. Furthermore, VDR activation has been reported to be involved in BA transport, metabolism, and detoxification through the stimulation of CYP3A [9]. A selective binding of LCA acetate to VDR was proposed to be 30 times better than LCA itself and had less specificity binding with FXR and PXR [47]. Vitamin D deficiency may increase the risk of inflammatory bowel disease and osteoporosis in individuals. Nowadays, Vitamin D deficiency is not just linked to bone- and gastrointestinal-tract-related disorders, but its deficiency has also been associated with several cardiovascular diseases (CVDs), such as coronary heart disease, heart failure, atrial fibrillation, stroke, peripheral arterial disease, endothelial dysfunction, and arterial stiffness [48,49,50].

Liver X receptor (LXR) is important in cholesterol homeostasis, particularly in BA metabolism, transport, and excretion. LXR is activated by endogenous oxysterols and oxidized derivatives of cholesterols. Increased level of oxysterols in cells activates LXR and protects cells from the effect of high cholesterol. Unfortunately, cholesterol/LXR signaling leading to upregulation of CYP7A1 gene and ATP-binding cassette (ABC) G5 and G8 have only been observed in animal models but not in the human liver [51,52]. Therefore, most studies have focused on cholesterol/FXR pathways rather than cholesterol/LXR activation pathways.

### 2.2. Takeda G-Protein-Coupled Receptor 5

Takeda G-Protein-Coupled Receptor 5 (TGR5) (GP-BAR 1 or M-BAR) was first discovered by Kawamata et al. [53]. Takeda G-Protein-Coupled Receptor 5 expression is expressed in different types of cells, such as endocrine glands, adipocytes, muscles, immune response, spinal cord, and the enteric nervous system. Takeda G-Protein-Coupled Receptor 5 was reported to suppress rabbit alveolar macrophages function in response to BAs (LCA, DCA, and CDCA) treatment and subsequently inhibit lipopolysaccharide (LPS)-induced tumor necrosis factor alpha (TNFα) secretion [53]. Takeda G-Protein-Coupled Receptor 5 has also been shown to be activated by taurolithocholic acid (TLCA). Takeda G-Protein-Coupled Receptor 5 is a type of G-protein-coupled receptors (GPCR) that requires activated G protein leading to cAMP accumulation [53] and signaling kinase proteins, such as protein kinase B (Akt) and ERK 1/2. Takeda G-Protein-Coupled Receptor 5 has been found to be highly regulated in the intestine. Takeda G-Protein-Coupled Receptor 5 signaling was shown to regulate the intestinal glucagon-like peptide-1 (GLP-1) release and subsequently improve the liver function of obese mice [54]. Glucagon-like peptide-1 is involved in glucose homeostasis, where it increases the insulin secretion and improves glucose tolerance. Takeda G-Protein-Coupled Receptor 5 is also known to protect liver injury by inhibiting LPS-induced cytokine expression in Kupffer cells [55]. A recent study showed that bile acid receptor TGR5 deletion in mouse macrophage increased in the liver. They found that TGR5–Akt–mTOR signaling pathway was important in improving insulin action and modulating chemokine expression in obesity-induced insulin-resistant models [56]. In the heart, TGR5 suppresses inflammation and reduces atheroma plaques formation and thus decreases the atherosclerosis effect [57]. In bovine aortic endothelial cells, activated TGR5 was shown to induce nitric oxide productions and inhibit nuclear factor kappa B (NF-κB) activity, which suppressed monocyte adhesion and prevented the accumulation of atheroma plaques in the arteries [57].

### 2.3. Muscarinic Receptor

Muscarinic (M) receptors are classified into M1, M2, M3, M4, and M5. Muscarinic receptors are divided into two categories according to the way in which they inhibit adenylate cyclase (M2 and M4) or stimulate phosphoinositide hydrolysis (M1, M2, and M5). Historically, the selective effect of muscarinic receptor in the brain has been suggested to be a therapeutic target for the treatment of Parkinson diseases due to the modulation of muscarinic antagonist receptor [58]. The muscarinic receptors of the eyes help in protecting tear film and lens for the treatment of myopia among children [59]. In the heart, muscarinic receptor stimulation by the parasympathetic nerves modulates contraction [60]. Cheng et al. reported that bile acid lithocholyltaurine is a ligand for M3 muscarinic receptor [61]. Binding of lithocholyltaurine to M3 receptor of Chinese hamster ovary (CHO) cells were shown to stimulate acetylcholine-induced inositol phosphate formation and mitogen-activated (MAP) kinase phosphorylation [24]. Taurocholate (TC) was shown to inhibit cAMP, affect calcium transient amplitude, and reduce contraction of cardiomyocytes (CMs). These effects were mediated by the M2 muscarinic receptor [62]. Similarly, Ibrahim et al. [63] showed that conjugated bile acids action was mediated by M2 muscarinic receptor and not by sphingosine 1 phosphate 2 receptor.

### 2.4. Sphingosine-1-Phosphate Receptor

Bile acid has been reported to regulate sphingosine-1-phosphate (S1P) level. Sphingosine-1-phosphate is known as the most potent substrate of sphingolipid. Sphingosine-1-phosphate pathways determine cell fate and whether it will undergo proapoptotic signaling or prosurvival signaling. There are five subtypes of S1P receptor, known as S1P1, S1P2, S1P3, S1P4, and S1P5 receptor. Sphingosine-1-phosphate receptors are GPCR; hence, each receptor subtype’s downstream actions are determined by the G protein that couple to its receptor. In hepatocytes, the most abundantly expressed S1P receptor are S1P1 and S1P2, which are able to activate ERK 1/2 and Akt pathways by the natural ligand for GPCR, i.e., S1P [26]. On the other hand, S1P1, S1P2, and S1P3 receptors are mostly abundant in the heart, whereas the expression of S1P4 and S1P5 are only limited to immune response and the nervous system. Sphingosine-1-phosphate 2 has been reported to be a receptor for conjugated bile acid TC, which activates ERK 1/2 and Akt signaling pathways [27]. Studer et al. [27] showed that S1P2 receptor antagonist JTE-013 significantly inhibits the hepatic ERK 1/2 and Akt activation, thus impeding the sphingosine kinase 2 (SphK2) production. Recently, S1P2 receptor signaling was reported to crosstalk with epidermal growth factor receptor (EGFR)-mediated signaling [64]. Sphingosine-1-phosphate 2 receptor was also shown to be highly expressed in mouse hepatocytes [18]. The study found that the mRNA level of the S1P2 receptor was highly regulated in primary hepatocytes culture and liver in an in vivo model. Taurocholic acid has also been shown to induce expression of S1P2 receptor and promote cholangiocarcinoma growth [28]. In the heart, S1P1 is important in angiogenesis and other important cardiac cellular mechanisms [29]. However, the function of BA-S1P1 receptor activity in the heart is not fully understood.

### 2.5. Large Conductance Voltage- and Ca^2+^-Activated Potassium (K^+^) (BK) Channels

Studies have shown that apart from known bile acid receptors (FXR, LXR, VDR, PXR, TGR5, muscarinic, and S1P), BAs also activate non classical receptor response, including large conductance voltage- and Ca^2+^-activated potassium (K^+^) (BK) channels. Bukiya et al. [65,66] reported that the LC activates BK channel through unique docking at β1 subunit transmembrane domain 2 (TM2) and enhances vascular myocytes BK channel activity [65]. High concentration of BA is required for the activation of BK channels compared to FXR or PXR. There are two possible actions of BAs in activating the ion channels: (1) direct binding of BA to the channel protein by inducing its conformational changes for gate opening and (2) BA interfering with the plasma membrane of the cells by altering its lipid surrounding, thus inducing sensitive channel opening [30]. In another study, BA was demonstrated to activate BK channel of patients with liver cirrhosis and increase the risk of cirrhotic cardiomyopathy development [31]. Meanwhile, in another study, taurine-conjugated hydrophobic bile acid was reported to have a negative inotropic effect, reducing the duration of the action potential and increasing outward potassium currents [67].

## 3. The Most Hydrophilic Bile Acids—Ursodeoxycholic Acid

Around 1000 years ago, traditional Chinese practitioners discovered that the liver extracted from black bear could treat chronic liver diseases. At that time, the bear bile prescription was very expensive due to its lack of availability in the market. For the past 50 years, scientists have elucidated that the liver of the black bear contains high levels of UDCA. Recently, UDCA was shown to reduce cholesterol absorption and dissolve the cholesterol formed in gallbladder as an alternative medication. In addition, another study proved that UDCA protects liver cells by reducing the elevated liver enzyme levels and facilitating bile flow [68].

Ursodeoxycholic acid (molecular formula = C_24_H_40_O_4_; molecular weight = 392.572 g/mol) is synthesized in the intestine by bacteria. CDCA and UDCA are different due to their hydroxyl group position in the steroid skeleton. Ursodeoxycholic acid hydroxyl group is positioned at β chair conformation cyclic, while CDCA is in α. The α isomer is less stable than the β isomer. Furthermore, UDCA has been shown to diminish the properties of hydrophobic bile acid-induced oxidative damage [69,70,71]. Commercially, synthetic UDCA is known as ursodiol. It is soluble in water and is manufactured as a white or off-white crystalline powder with a bitter taste.

Ursodeoxycholic acid has been reported to be beneficial for the treatment of colorectal adenoma [72] and inflammatory bowel disease [73]. In liver and liver-related diseases, UDCA has been shown to improve biliary secretion in primary sclerosing cholangiocytes and stimulate detoxification of hydrophobic bile acids in primary biliary cirrhosis. The upregulation of efflux pumps expressions such as MRP2, BCRP (breast cancer transporter resistance protein), and P-glycoprotein in cells treated with UDCA. This shows that UDCA is important for small intestinal detoxification [74]. In primary rat hepatocytes, UDCA was shown to downregulate p53 expression and prevent apoptosis signaling through reduction of Bcl- 2(B-cell lymphoma 2) associated X protein (Bax) expression, mitochondrial translocation, and cytochrome c release [14]. Clinical studies showed that UDCA intervention was able to restore the liver function of obstructive jaundice patients after endoscopic treatment [75]. Intrahepatic cholestasis (ICP) is a pregnancy liver disorder that is reported to affect pregnancy during the third trimester and is mostly treated with UDCA. In ICP, the elevated serum bile acids can lead to maternal pruritus and increase of the fetal bile acid constituent. UDCA is proven to decrease the toxicity effect of these elevated hydrophobic bile acids. Clinical studies have shown a reduction in pruritus during pregnancy with UDCA treatment [76]. Moreover, studies have shown that UDCA relieves the effect of ICP by elevating the hepatic intracellular secretion [77].

Ursodeoxycholic acid dissolves gallstone formation and prevents related disease such as chronic cholecystitis, biliary colic, pancreatitis, or obstructive jaundice. The nonsurgical dissolution of gallstone by UDCA is recommended as a safe and effective method compared to laparoscopic cholecystectomy operation. UDCA also tends to be more potent than chenodeoxycholic acid as a desaturating agent for dissolving gallstone in non-obese patients [78]. In cholesterol gallstone patients, UDCA was shown to improve the contraction of gallbladder smooth muscle strips after six weeks of administration compared to normal patients, thus suggesting that the administration of UDCA for longer periods increases the effectiveness of gallbladder contraction [79]. Currently, UDCA is not used for other disorders or diseases and is still under consideration despite numerous studies having reported its wide therapeutic effects.

## 4. Mechanism of Action by Ursodeoxycholic Acid

Ursodeoxycholic acid mechanisms of action could be divided into antiapoptotic and proapoptotic signaling. In the hepatocytes, UDCA was shown to inhibit Fas (First apoptosis signal)-ligand-induced apoptosis in mouse hepatocytes and was postulated to act through mitochondria membrane permeability [80]. Ursodeoxycholic acid is a promising drug to treat liver-related diseases in the future as it has been shown to play a significant role in cholestasis, fibrosis, and sclerosis. Moreover, UDCA has been shown to inhibit the effect of GCDCA-induced apoptosis that is independent of caspase-8/caspase-3/caspase-9 and dependent on antiapoptotic kinases, for example, p38 mitogen-activated protein kinase (p38), ERK 1/2, and phosphotidyl inositol-3 kinase (PI3K) [81]. Apart from the liver, UDCA has also been reported to have antiapoptotic effect in other cells, such as cholangiocytes [82], brain [83], eyes [84], and osteosarcoma fibroblast. At the same time, numerous studies have shown that apart from antiapoptotic signaling, UDCA also play a role in proapoptotic signaling in cancer cells, such as gastric, prostate, and colon cancer cells.

In hepatocytes, UDCA has been shown to counteract the effect of more hydrophobic bile acid and toxic compound secretion. These responses are thought to be mediated by protein kinase C alpha (PKCα) and intracellular calcium [Ca^2+^]_i_. Murakami et al. [85] suggested the beneficial effect of UDCA as a potent drug to help reduce blood glucose and stimulate GLP-1 in type 2 diabetes mellitus patients. UDCA has also been suggested to decrease the number of active macrophages, thus contributing to the mechanisms of cytoprotection [86]. In another study, UDCA was shown to be cytoprotective against hydrophobic bile acids by inhibiting the formation of ROS and translocation of the pro-apoptotic protein Bax from cytosol to mitochondria. Ursodeoxycholic acid was shown to stabilize the mitochondria membrane by activating the glucocorticoid receptor in an in vitro model of Parkinson’s disease [87]. Apart from that, UDCA’s effect on CMs [62] and osteosarcoma fibroblast has also been reported [88]. Ursodeoxycholic acid has been shown to inhibit the effect of bilirubin- and LCA-induced apoptosis in osteoblastic cells by preventing translocation of pro-apoptotic protein Bax to mitochondria and downregulating caspase-3 activity.

## 5. Ursodeoxycholic Acid Protects the Heart against More Hydrophobic Bile Acids

In the heart, Williamson et al. (2001) was the first to demonstrate that the primary bile acid TC affects CM contraction and alters their intracellular calcium ([Ca^2+^]_i_) dynamics [89]. Moreover, the effect of TC on CMs has been shown to be different in single CM and monolayer CMs. The differences include the beating rate, amplitude of contraction, and desynchronization of [Ca^2+^]_i_ dynamics [90,91]. In embryonic stem cell-derived CMs, TC has been shown to cause abnormal Ca^2+^ dynamics in the early but not in the late stages of cells [92]. Taurocholic acid is a taurine-conjugated hydrophobic BA that has been reported as a partial agonist of the muscarinic M2 receptor (Gαi-coupled receptor), which induces arrhythmia in cultured CMs [62]. In agreement with Sheikh Abdul Kadir et al. [62], Mohamed et al. [93] reported that UDCA maintains the normal intracellular [Ca^2+^]_i_ dynamics against hypoxic condition, which is also mediated by Gαi-coupled receptor. Gorelik et al. [94] showed that UDCA protects the heart from TC-induced arrhythmias by improving contraction and calcium dynamic changes in CMs. In addition, the study suggested that the cardioprotective effect of UDCA is similar to dexamethasone, which alters the expression of genes for bile acid transporter and metabolism in CMs. Dexamethasone has been reported in protecting the contraction rate of CMs during arrhythmia. In another study, UDCA protection of CMs against TC arrhythmogenic consequences was suggested to be mediated by the adenosine triphosphate-gated K^+^ (K^ATP^) channels and [Ca^2+^]_i_ [91]. In addition, UDCA, the most hydrophilic bile acid, has been shown to protect cholestasis fetal heart model from BA-induced arrhythmia [89,90,91]. In liver cirrhosis patients, an increase in hydrophobic BAs causes QT interval prolongation and ventricular arrhythmia [31]. Consequently, these liver cirrhosis patients may develop cirrhotic cardiomyopathy [67]. The effect of BA is suggested to occur here through the BK channel. Although the finding is not completely in agreement with Miragoli et al. [91], both results suggest that BA action could be mediated by K^+^ channel. As for the [Ca^2+^]_i_, BAs have been widely reported to alter calcium dynamics in different types of cells. In isolated hepatocytes culture, bile acids was shown to cause increase in cytosolic calcium level, upregulation of [Ca^2+^]_i_ expression involving ASBT transporter in cholangiocytes, trigger the release of [Ca^2+^]_i_ from inositol phosphate (IP3)-sensitive endoplasmic reticulum, and trigger large calcium conductance to activate the potassium channels [95,96,97,98]. These responses are postulated to be mediated by the TGR5. The LCA–TGR5 binding induces intracellular cAMP, which regulates various biological processes [99]. However, this is not seen in all cell types, for example, it was not observed in human platelets or neuroblastoma cell lines (NG108-15) [100]. Therefore, the effect of bile acids on calcium signals has been suggested to be cell-specific.

There are two isoforms of LXR, known as LXRα and LXRẞ. Both isoforms have been reported to be involved in cardiovascular diseases and atherosclerosis reduction. Song et al. (2000) reported that taurine-conjugated UDCA activates the LXR-binding response elements (LXRE) in the CYP7A1 promoter to induce LXR activation and promote CYP7A1 activity [51]. Bradley et al. (2007) reported that the LXR activation reduces formation of atherosclerosis lesion [100]. This is expected as LXR is known to regulate bile acid synthesis through the LXRE on CYP7A1, which is the rate-limiting enzyme of the cholesterol pathway. However, the BAs/LXR route of signaling has not been further investigated after a few studies demonstrated that this route of signals was important in animal models but not in humans.

## 6. Past, Current, and Future Perspectives on Role of Ursodeoxycholic Acid in Cardiovascular Diseases

Heart disease may cause CM injury or loss. Cardiomyocytes play a major role in maintaining the physiology of the heart. Sufficient supply of oxygenated blood throughout the coronary arteries protects heart muscle and improves the pumping ability of the heart. Ursodeoxycholic acid has been reported to play an important role in heart diseases, with many studies further proving the potential of UDCA as a future prophylaxis for CVDs (Table 2).

In 1998, Bährle and co-workers reported a significant improvement in acute rejection episodes in heart transplant patients treated with UDCA compared to the untreated ones [109]. However, the study by Bärhle was done retrospectively. Therefore, the cardioprotection mechanism in heart transplant is not fully understood. In ischemia–reperfusion injury, Lee et al. [101] have shown that UDCA reduces lactate dehydrogenase release and enhances the recovery of cardiac contractile function during reperfusion. In addition, UDCA protection against reperfusion injury in rat myocardium was suggested to act by inhibiting the mitochondrial permeability transition pore (MPTP) dependent on PI3K/Akt pathway [102]. Rajesh et al. [102] further showed that UDCA protected in vivo and in vitro models of ischemia–reperfusion injury by mediating the phosphorylation of Bcl-2-associated death (Bad) protein and preventing its translocation to mitochondria, thus blocking the downregulation of Bcl-2 and the opening of MPTP. In patients with heart failure, UDCA treatment has been shown to improve endothelium- and NO-independent vasodilatation, which maintains the arterial flow of impaired nitric oxide production [107]. In another clinical study, patients with chronic heart failure received 500 mg of UDCA twice daily for four weeks, and UDCA was observed to improve post ischemia peripheral blood flow in the arms and legs of patients. Apart from that, liver function was improved, with the levels of γ-glutamyl transferase, aspartate transaminase, and soluble tumor necrosis factor-α receptor 1 (TNF-1α) lower in post-treatment compared to pretreatment and control subjects [108].

**Figure 2:**
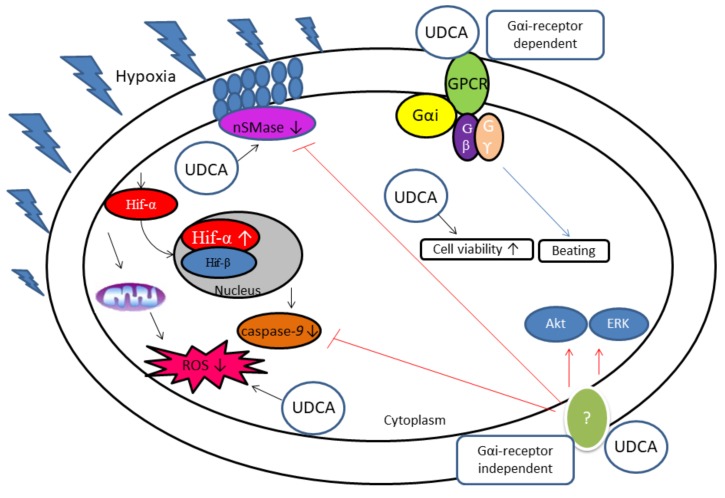
Proposed anti-apoptosis mechanism for UDCA cardioprotection against hypoxia. Pertussis toxin does not block the effect of UDCA on caspase-9, neutral SMase expression, ROS production, and Hif-1α expression in hypoxia-induced CMs. Beating rate is partially inhibited by PTX, suggestive of nonsensitive PTX pathway (pathways independent of Gα_i_-coupled-receptor) involvement. Ursodeoxycholic acid cardioprotection has been reported to regulate the activation of survival signaling proteins (ERK 1/2 and Akt) and neutral SMase in hypoxia-induced CMs [104]. UDCA has been shown to downregulate caspase-9 protein expression and neutral SMase activity and upregulate phosphorylation of ERK 1/2 and Akt via Gαi-independent pathways (red line) to promote cardioprotection against the effects of CoCl_2_. Meanwhile, UDCA has been shown to inhibit Hif-1α, ROS production, and caspase-9 protein expressions in CoCl_2_-induced hypoxia CMs [91]. The data also suggests that UDCA cardioprotection in CoCl_2_-induced hypoxia could be mediated through dependent Gαi pathways on CM beating rate (blue line). GPCR: G-protein-coupled receptor; Gαi, Gi alpha subunit is a G protein subunit that inhibits cAMP from production; Gβ, G-beta; Gγ, G-gamma; nSMase, neutral sphingomyelinase; UDCA, ursodeoxycholic acid; Hif-α, hypoxia inducible factor alpha; Hif-ẞ, hypoxia inducible factor beta; ROS, reactive oxygen species; Akt, protein kinase B; ERK, extracellular signal regulated kinase; PTX, pertussin toxin.

In 2013, a study reported UDCA as an FXR agonist and that the UDCA-FXR signals may led to the inhibition of NO synthase expression. As a consequence, it would lower the risk of congestive heart failure and other CVDs [110]. Recently, Mahmoud and Elshazly [103] demonstrated that UDCA reduced uric acid level and improved insulin resistance in rats with fructose-induced metabolic syndrome. Studies have shown that UDCA upregulates survival signaling protein ERK 1/2 and Akt [104] and downregulates caspase-9 and ROS generation in CoCl_2_-induced hypoxic CMs [105]. A summary of the findings from these two studies is given in Figure 2. Increased hydrophobic BAs have been associated with cardiac hypertrophy, CMs apoptosis, and abnormal cardiac hemodynamics [111]. Recently, a clinical study reported that the total bile acid level is associated with the enlargement of left atrial volume and interferes with the hyperdynamic circulation in patients with cirrhosis [112]. Hydrophobic BAs have been shown to increase the risk of atherosclerosis development and other CVDs through the inhibition of endoplasmic reticulum stress, which interrupts lipogenic pathways, free cholesterol-induced cell death in macrophages [113], reduction of major histocompatibility complex (MHC)-associated antigen presentation [114], and diminishing of the inflammatory environment [111]. In contrast, the hydrophilic BA UDCA was shown to exert anti atherogenic activity in diabetic atherosclerosis mouse model through reduction of endoplasmic reticulum stress, receptor for advanced glycation endproduct (RAGE) signaling, and proinflammatory responses, including ROS production and Nf-κB activation [106].

In 2012, Von Haehling et al. (2012) reported that UDCA improved peripheral blood flow in chronic heart failure patients [108]. The study suggested that UDCA may work due to its effects on gut microbiota. The idea is in agreement with another study [115], which demonstrated that UDCA and its conjugates normalized the gut microbiota firmicutes/Bacteroidetes ratio as this ratio is increased in mice with colitis. This gut microbiota ratio is also used to indicate gut health in humans [116,117]. Nowadays, probiotic supplements have been shown as a beneficial prophylaxis to lower the risk of CVDs, such as ischemic heart disease [118], post infarction myocardial hypertrophy failure [119], and chronic systolic heart failure [120]. Although not much attention has been given to the potential of UDCA in lowering the risk of CVDs, it is worthy of future studies in order to understand the effects of UDCA on the probiotic profile of CVD models and patients.

Ursodeoxycholic acid has been previously shown to inhibit colon carcinoma development mediated by sphingomyelinase (SMase) [121]. In unpolarized colon cancer, UDCA is reported to induce alkaline SMase, which inhibits cells proliferation. Neutral SMase activation is detected in post myocardial infarction of chronic heart failure (CHF) patients, and inhibition of neutral SMase activity leads to failure of recovery in the hearts of post infarcted patients. In the heart, an abundance of sphingomyelin in the cells promotes atherogenesis and may lead to angiographic coronary heart disease (CHD) and left ventricular systolic dysfunction [122]. In addition, secretory acid SMase has been shown to be upregulated in CHF patients, with the upregulation of this enzyme reported to be associated with loss of functional capacity in skeletal muscle [123]. Furthermore, CHF patients were recorded to express 90% more of total SMase compared to healthy patients. Additionally, the study found that high total SMase in CHF leads to functional and structural impairment of skeletal muscle tissue. Hence, it is crucial to find whether UDCA is able to affect the SMase expression level in the heart disease model. Hanafi et al. [104] were the first to report that UDCA protects CMs against CoCl_2_-induced hypoxia. The study reported that in hypoxic in vitro heart model, UDCA upregulated Akt, ERK, and neutral SMase expression. Furthermore, the findings proposed that UDCA cardioprotection effects are due to its ability to regulate anti-apoptosis mechanism as summarized in Figure 2. This was in agreement with the results reported by Empinado et al. (2014), which demonstrated high accumulation of neutral SMase, ceramide, and S1P but no changes in acid SMase and sphingosine activity in CHF rat models [122]. In addition, another study reported that neutral SMase activation is highly elevated in hypoxia-reoxygenated CMs [124].

**Figure 3:**
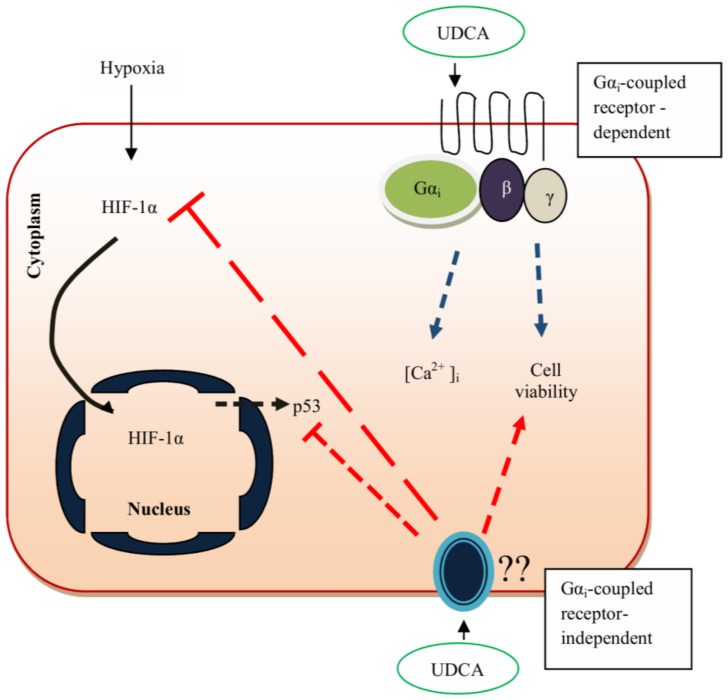
Proposed mechanisms for UDCA cardioprotection in maintaining normal [Ca^2+^]_i_ and HIF-1α level. Binding of UDCA to PTX-sensitive receptor partially improves cell survival. However, PTX does not block the effect of UDCA on [Ca^2+^]_i_, HIF-1α translocation, and p53 protein expression against hypoxia. Cell viability is partially inhibited by PTX; dual pathway is suggested to be involved (Gα_i_-coupled receptor-dependent and -independent pathways). Blue arrow, Gα_i_-coupled receptor-dependent pathways; red arrow, Gα_i_-coupled receptor-independent pathways. (UDCA, ursodeoxycholic acid; Gαi, G-alpha; ẞ, G-beta; γ, G-gamma; Hif-1 alpha, hypoxia inducible factor 1 alpha; [Ca^2+^]_i_, intracellular calcium; p53, cellular tumor antigen p53).

Sphingomyelinases are suggested as important chemicals in heart cells and their regulation determines the level of ceramide in the cells [125,126,127]. Ceramide is a by-product of SMases enzyme activity and is known to be involved in cascade of pathways, such as apoptosis and stress responses. It is grouped as components of CHF. Moreover, its level is regulated by the secreted S1P, which binds to GPCR–S1P receptor in rat hepatocytes. Conjugated bile acid has been shown to promote cytoprotective effect through S1P1 activation, ERK, and Akt signaling pathways [109]. In the heart, S1P1 is abundantly expressed, and S1P receptors regulate cardiac physiology and pathophysiology. S1P1 receptor is found exclusively bound to Gi-sensitive protein-coupled receptor [127]. Sphingosine-1-phosphate 1 expression activates the inhibition of cAMP formation and antagonizes adrenergic receptor-mediated contractility. Meanwhile, S1P2 receptors activity in cardiac myocytes mediates cardioprotection by activation of Rho. In addition, S1P3 receptors are expressed in the heart, and its activation results in bradycardia [127]. Mohamed et al. [93] reported that UDCA’s protection of CMs against hypoxia could be mediated partly by S1P1 receptor. The study suggested that cardioprotection of UDCA was observed to be similar to FTY720 (S1P receptor agonist) in inhibiting the lethal effect of hypoxia through inhibition of HIF-1α and p53 protein and restoration of normal [Ca^2+^]_i_ in hypoxic CMs [93]. As demonstrated by multiple pieces of evidence, BAs activate various pathways, as reviewed in other sections, and UDCA is suggested to activate Gαi-coupled receptor-dependent pathways and/or Gαi-coupled receptor-independent pathways in mediating cellular response. In summary, the data reported from this study suggest that UDCA protects CMs by inhibiting the expression of HIF-1α and p53 protein via Gαi-coupled receptor-independent pathways (Figure 3; red arrow). Therefore, UDCA improves CM viability, and [Ca^2+^]_i_ can be mediated by Gαi-coupled receptor pathways (Figure 3; blue arrow). The data also suggest that the UDCA cardioprotection against the effect of hypoxia on CMs viability might work through both Gαi-coupled receptor-dependent and -independent pathways.

## 7. Conclusions

Bile acid signaling via receptor-dependent (FXR, LXR, VDR, PXR, TGR5, S1P, Muscarinic) and channel-mediated (BK channel) mechanisms have been widely studied on different types of cells. The role of BAs as signaling molecules in liver diseases, such cirrhosis and interahepatic cholestasis of pregnancy, are well known. This paper provided a general overview of BAs as ligands mediated by these reported receptors and channels, with a focus on heart cells and on how UDCA cardioprotection has been discovered in recent research. Bile acids has been shown to possess both apoptotic and antiapoptotic signaling mechanisms, which are suggested to be cell-type dependent. Most BAs and UDCA actions seem to be dual-receptor/channel ligand. Based on past and present findings, cardioprotection of UDCA could be partially mediated by GCPR type but not muscarinic receptor. Hence, most studies agree that BAs’ dual-receptor/channel activation are due to their abilities to act as hormones and regulatory ligands. In the heart, UDCA was first demonstrated to protect the heart cells against acute allograft rejection after transplantation and ischemic–reperfusion injury. After about 10 years, a few more studies showed that UDCA protects neonatal CMs against the effect of hydrophobic bile acid taurocholate, which is raised in maternal and fetal serum during cholestasis of pregnancy and may cause fetal dysrhythmia. In fetal heart and neonatal CM cultures, bile acid transporters are expressed at relatively low level, which suggests that UDCA may not act through a classical pathway as it does in the liver and small intestine. Several lines of evidence have demonstrated the involvement of UDCA with the sphingosine-1-phosphate (S1P) receptor (Gαi-coupled receptor) in hepatocytes, but not in the heart. Although a lot of studies on UDCA have demonstrated the potential use of UDCA to overcome myocardial injury, the cardioprotection mechanism of UDCA is still not well understood. In clinical trials, UDCA seems to improve the cardiac health of CVD patients. However, to further confirm the importance of UDCA in humans, more comprehensive studies with more number of patients are needed. Vast information on the applications of UDCA using preclinical and clinical studies should be made available in the future for better comparison and understanding of the subject.

## Figures and Tables

**Table 1:** The main receptor involved in bile acids signaling.

Receptor	Tissue	Bile Acid	Summary of Implications of High BA Pool	References
FXR	Liver, cholangiocytes, colonocytes, small intestine, heart	CDCA, DCA, LCA	Glucose metabolism and cholesterol metabolism are altered, which result in downregulation of LDL-R expression, increase in LDL-C levels, and upregulation of transcriptional activity of bile acids.	[14,15,16,17]
Others; PXR, LXR, VDR, S1P	Liver and heart	LCA	The receptors regulate hepatic lipid metabolism, activate ERK 1/2, and Akt and then lead to regulation of lipid and glucose metabolism.	[18,19,20,21]
TGR5	Liver, heart, dendritic cells,	TLCA, LCA, DCA, CDCA, CA, UDCA,	Modulates insulin signaling pathway and aids in the regulation hepatic glucose metabolism and inhibition of LPS-induced cytokine expression.	[13,22,23]
Muscarinic	Liver, brain, eyes, heart, and colon carcinoma	Lithocholyltaurine (LCT), TCA	Modulate glucose homeostasis, thermogenesis, inflammatory response, and stimulate parasympathetic nerves.	[24,25]
Sphingosine-1-phospahate (S1P)	Cholangio carcinoma, heart, liver	TCA	Promotes cholangiocarcinoma growth, lipid metabolism, angiogenesis, and cardiac cellular signaling.	[26,27,28,29]
Large conductance voltage- and Ca^2+^-activated potassium (K+) (BK) channels	Liver and intestinal tract	LCA	Improves vascular muscle cells vasodilation.	[30,31]

BA, bile acid; CDCA, chenodeoxycholic acid; DCA, deoxycholic acid; LCA, lithocholic acid; TLCA, taurolithocholic acid; TCA, taurocholic acid; CA, cholic acid; UDCA, ursodeoxycholic acid; ERK, extracellular signal-regulated kinase; FXR, farnesoid X-receptor; LDL-R, Low-density lipoprotein- receptor; LDL-C, Low-Density lipoprotein- cholesterol; PXR, Pregnane X receptor; LXR, Liver X receptor; VDR, Vitamin D receptor.

**Table 2:** The summary of UDCA mechanisms of action with the experimental models/subjects and concentrations used.

Model	Suggested UDCA Mechanism of Action	Concentration of UDCA Used	References
In vitro rat model of the fetal heart	UDCA induces cAMP release without any effects on contraction rate, which is mediated through TGR5.	100 µM	[63]
In vitro rat model of the cholestatic fetal heart	UDCA activates K_ATP_ channels and improves intracellular calcium level.	100 µM	[91]
In vitro rat model of ischemia–reperfusion	UDCA reduces LDH release and enhances the recovery of cardiac contractile function during reperfusion.	80–160 µM	[101]
In vitro and in vivo rat models of ischemia–reperfusion	UDCA inhibits the opening of MPTP and Bcl-2 via PI3K/Akt pathway.	40 mg/kg	[102]
In vivo rat model of metabolic syndrome	UDCA reduces uric acid level and improves insulin resistance of fructose-induced metabolic syndrome rat.	150 mg/kg	[103]
In vitro rat model of hypoxic cells	UDCA inhibits HIF-1α expression, upregulates ERK 1/2, and Akt while downregulating caspase-9 and reactive oxygen species (ROS) generation in cobalt chloride (CoCl_2_)-induced hypoxic CMs.	100 µM	[93,104,105]
In vivo mouse model of diabetic atherosclerosis	UDCA exerts antiatherogenic activity through reduction of endoplasmic reticulum stress, receptor for advanced glycation end product (RAGE) signaling, and proinflammatory responses of ROS and Nf-κB.	100 µM	[106]
Patients with coronary heart disease	UDCA improves endothelium- and NO-independent vasodilatation that maintains the arterial flow in patients with heart failure.	13–19 mg/kg	[107]
Patients with chronic heart failure	UDCA improves liver function and lowers the level of γ-glutamyl transferase, aspartate transaminase, and soluble TNF-α receptor 1.	1280 µM	[108]

UDCA, ursodeoxycholic acid; cAMP, cyclic adenosine monophosphate; TGR5, Takeda G-protein-coupled bile acid receptor 1; K^ATP^, ATP-sensitive potassium channel; LDH, lactate dehydrogenase; MPTP, mitochondrial permeability transition pore; Bcl-2, B-cell lymphoma 2; PI3K, phhosphoinositide 3-kinase; HIF-1α, hypoxia inducible factor 1 alpha; ERK 1/2, extracellular regulated kinase; Akt, protein kinase B; CoCl_22_, cobalt chloride; CMs, CMs; ROS, reactive oxygen species; Nf-κB, nuclear factor kappa B; NO, nitric oxide; TNF-α receptor 1, tumor necrosis factor 1.
